# Respiratory Cancer and Inhaled Inorganic Arsenic in Copper Smelters Workers: A Linear Relationship with Cumulative Exposure that Increases with Concentration

**DOI:** 10.1289/ehp.11515

**Published:** 2008-07-23

**Authors:** Jay H. Lubin, Lee E. Moore, Joseph F. Fraumeni, Kenneth P. Cantor

**Affiliations:** 1 Biostatistics Branch, Division of Cancer Epidemiology and Genetics, National Cancer Institute, Rockville, Maryland, USA; 2 Occupational and Environmental Epidemiology Branch, Division of Cancer Epidemiology and Genetics, Rockville, Maryland, USA; 3 Office of the Director, Division of Cancer Epidemiology and Genetics, National Cancer Institute, Rockville, Maryland, USA

**Keywords:** arsenic, dose, response relationship, lung neoplasms, occupational diseases

## Abstract

**Background:**

Inhalation of high levels of airborne inorganic arsenic is a recognized cause of respiratory cancer. Although multiple epidemiologic studies have demonstrated this association, there have been few analyses of the mathematical relationship between cumulative arsenic exposure and risk of respiratory cancer, and no assessment as to whether and how arsenic concentration may modify this association.

**Objectives:**

The objective is an evaluation of the shape of the relationship between respiratory cancer mortality and cumulative inhaled arsenic exposure among copper smelter workers, and the modification of that relationship by arsenic concentration.

**Methods:**

We used Poisson regression methods to analyze data from a cohort of arsenic-exposed copper smelter workers under a linear-exponential model for the excess relative risk.

**Results:**

Within categories of arsenic concentration, the association between respiratory cancer and cumulative arsenic exposure was consistent with linearity. The slope of the linear relationship with cumulative exposure increased with increasing arsenic concentration category.

**Conclusions:**

Our results suggested a direct concentration effect from inhaled inorganic arsenic, whereby the excess relative risk for a fixed cumulative exposure was greater when delivered at a higher concentration and shorter duration than when delivered at a lower concentration and longer duration.

The International Agency for Research on Cancer (1980, 1987) identifies inhalation of inorganic arsenic as a cause of lung cancer in humans. The evidence has come primarily from epidemiologic studies of miners and smelter workers who inhaled high levels of inorganic arsenic in airborne dust. Although increased risk of lung cancer from inhaled arsenic appears related to direct effects on the respiratory tract, there may be a systemic effect as well, because ingestion of high amounts of inorganic arsenic in drinking water has been associated with lung cancer mortality at levels comparable with occupational risks from inhaled arsenic (Bates et al. 1992; Lubin et al. 2000). Information on the mathematical relationship between cumulative inhaled arsenic exposure and respiratory cancer mortality is limited. Although a comparative evaluation of several occupational cohorts, mostly smelter workers, suggested a concave relationship between respiratory cancer mortality and cumulative arsenic exposure (Hertz-Picciotto and Smith 1993), a subsequent update of one large study revealed that its apparent nonlinearity was an artifact of exposure assessment and that relative risk (RR) trends with cumulative arsenic exposure were consistent with a linear relationship (Lubin et al. 2000). Previous analyses have focused on cumulative arsenic exposure and the shape of the exposure–response relationship, without considering the possible influence of arsenic concentration.

In the present analysis, we applied a novel methodology used previously in a study of smoking-related lung cancer (Lubin and Caporaso 2006) to model cumulative arsenic exposure and arsenic concentration. In particular, we examined the consequences of exposure “delivery,” that is, whether the RR of respiratory cancer at a fixed cumulative exposure delivered at higher arsenic concentration for shorter duration was greater than, equal to, or less than the RR from an equal cumulative exposure delivered at lower concentration for longer duration. We refer to these patterns as direct (increasing), constant (no variation), or inverse (decreasing) “exposure rate” effects.

Standard analyses of joint RRs by duration of arsenic exposure and arsenic concentration with nonexposed individuals as the referent group are problematic for characterizing the effects of arsenic concentration, because cumulative exposure varies. For illustration, consider duration of cigarette smoking, smoking intensity (cigarettes smoked per day), and total pack-years (years of smoking times cigarettes per day divided by 20). In a standard log-linear RR regression model that includes duration of smoking and cigarettes per day, the intensity parameter represents the ln(RR) per cigarette per day at a fixed duration. Interpretation of the duration parameter is similar but at a fixed intensity. A comparison of RRs at two different intensities reflects not only different intensities, but also different total pack-years. For example, at 30 years’ duration, RRs at 20 and 30 cigarettes per day reflect different intensities and different total pack-years—namely, 30 and 45 pack-years, respectively. Thus, RR patterns with duration and intensity cannot be viewed as “independent” because of the intrinsic confounding of pack-years. In contrast, in a model that includes pack-years and cigarettes per day, the parameter for cigarettes per day represents the comparison of risk for exposure delivered at lower intensity for longer duration with risk for an equal total exposure delivered at higher intensity for shorter duration (Lubin and Caporaso 2006). We use this framework to analyze inhalation of airborne inorganic arsenic in a cohort mortality study of smelter workers conducted in Montana (Lee and Fraumeni 1969; Lee-Feldstein 1983; Lubin et al. 1981, 2000).

Our goal of modeling the cumulative airborne arsenic exposure and arsenic concentration is to estimate a descriptive function that defines the modifying effect of concentration on the association between cumulative exposure and respiratory cancer. This effect modification is conceptually similar to the “dose rate effective factor” used in radiation epidemiology that describes for a specific type of radiation the effects on disease risk of total radiation dose delivered at lower (protracted or highly fractionated) dose rates compared with the effects at higher (or acute) dose rates (National Research Council 2006).

## Materials and Methods

### Data

The cohort study enrolled workers employed at a Montana copper smelter for ≥1 year before 1957, with follow-up starting 1 year after initial employment or 1 January 1938, whichever was later (Lee and Fraumeni 1969). The present analysis included 8,014 workers followed through 1989 (Lubin et al. 2000). We determined vital status from the National Death Index (http://www.cdc.gov/nchs/ndi.htm) and other sources, with causes of death recorded from death certificates. A total of 4,930 (62%) workers were deceased. Following previous analyses of this cohort, we consider respiratory cancer mortality as the primary outcome.

In the 1960s, investigators ranked each work site from 1 to 10 for its potential arsenic exposure and classified each site as “heavy” (ranks 8–10), “medium” (ranks 4–7), or “light” (ranks 1–3) arsenic exposure areas (Lee and Fraumeni 1969). We classified unspecified or unknown work areas as light exposure areas. We subsequently updated employment information through September 1977 (Lee-Feldstein 1986). Using 702 measurements of airborne arsenic from 1943 through 1958 and estimates of workers’ exposure times, investigators computed time-weighted average arsenic concentrations for light, medium, and heavy arsenic-exposed areas as 0.29, 0.58, and 11.3 mg/m^3^, respectively (Morris 1978). For each year of follow-up, we computed a cumulative exposure index in mg/m^3^-year, denoted *d*, as the product of years worked in light (L), medium (M), and heavy (H) exposure areas and the corresponding concentration—that is, *d* = 0.29 × L + 0.58 × M + 1.13 × H—where we down-weighted heavy exposure areas by a factor of 0.1 to reflect use of air filtration masks. This *a priori* choice of weight was similar to an empirically derived, risk-based estimate of weight (Lubin et al. 1981).

Many workers had recorded ages at last employment of < 40 and 50 years. Because there was no information on exposures after the workers left the smelter, we repeated analyses using person-years and respiratory cancer deaths in current workers, recent former (< 5 years) workers, and workers with last employment at ≥ 50 years of age to minimize effects of unmeasured exposures. The restricted subset included 261 respiratory cancer cases and 144,851 person-years. The number of respiratory cancer cases differed from a previous report (*n* = 252) (Lubin et al. 2000) because of a modified definition of the restrictions.

### Statistical analysis

We assumed survival time follows a piecewise exponential distribution and applied Poisson regression methods. We summarized deaths from respiratory cancer and person-years of follow-up in a multiway contingency table defined by attained age (nine levels: < 45, 45–49, . . . ,75–79, ≥ 80), calendar year (10 levels: 1935–39, . . . ,1985–90), cumulative arsenic exposure (mg/m^3^-year; 18 levels: < 0.25, 0.25–0.49, 0.50–0.74, 0.75–0.99, 1.0–1.4, 1.5–1.9, 2.0–3.9, . . . , 22–24.9, ≥25), mean arsenic concentration (mg/m^3^; eight levels: 0, 0.01–0.29, 0.3–0.39, 0.4–0.49, 0.5–0.59, 0.6–0.79, 0.8–0.99, ≥1.0), years since last arsenic exposure (three levels: < 5, 5–14, ≥ 15), and place of birth (two levels: U.S. or foreign born). For each cell of the cross-classification, we computed person-year–weighted means for the cross-classification variables. Because all workers were exposed to arsenic, we used age-specific and calendar-year-specific U.S. mortality rates for respiratory cancer in white males as a nonexposed referent population (Breslow and Day 1987).

We defined indicator variables *c_i_*, *i* = 1, . . . , *I*, for *I* concentration categories where *c_i_* = 1 for intensities within category *i* and zero otherwise, and modeled disease rate *r*(*x*,*d*,*c*) as


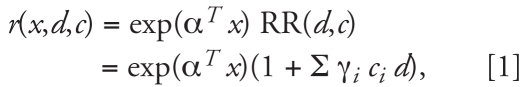


where *d* is cumulative arsenic exposure and *c* is arsenic concentration. The vectors of variables *x* and parameters α, where *T* denotes vector transpose, describe disease rates in non-exposed workers. Within concentration category *i*, RRs are linear in *d*; that is, RR(*d*,*c*) = 1 + γ*_i_* *d*, where γ*_i_* is the slope of the linear exposure–response, or ERR/mg/m^3^-year (ERR, excess relative risk). We used Wald-type confidence intervals (CIs) for γ*_i_* after reparameterizing γ*_i_* as exp(γ_i_*) to avoid range restrictions. Factoring out γ_1_, we rewrote RR(*d*,*c*) as


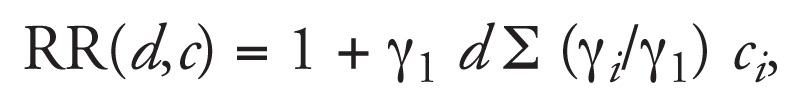


which suggested the following model for continuous concentration:


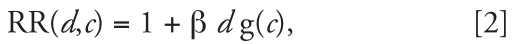


where β defined ERR/mg/m^3^-year at *g*(*c*) = 1. The *g*(.) function represents the modifying effects of arsenic concentration on the exposure–response relationship. Preliminary analysis suggested using the power form *g*(*c*) = exp{φ ln(*c*)}, where φ > 0, φ = 0, or φ < 0 denotes a direct (increasing), constant (no variation), or inverse (decreasing) concentration effect, respectively. Model fit did not improve with the addition of *c* or ln(*c*)^2^.

We extended model 1 to


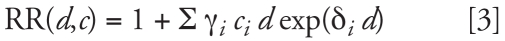


to evaluate departure from linearity, where δ*_i_* reflects concavity (δ*_i_* < 0) or convexity (δ*_i_* > 0) of the ERRs with cumulative exposure for the *i*th concentration category. The null hypothesis δ*_i_* = 0 specified no departure from linearity. We applied a similar extension to model 2.

Because all workers were exposed, we included the natural logarithm of the age- and year-specific respiratory cancer rates of U.S. males as a fixed offset variable (*x*) to represent rates in nonexposed workers. The model thus represented a regression equation for standardized mortality ratios (SMR) (Breslow and Day 1987). Although model 2 reflected relative SMRs, we refer to quantities as RRs. For analyses, we used the Epicure suite of programs (Preston et al. 2004).

## Results

The person-year–weighted mean cumulative arsenic exposure was 3.7 mg/m^3^-years, with a crude respiratory cancer mortality rate of 1.73 per 1,000 person-years, based on 446 total respiratory cancer deaths (Table 1). The SMR for respiratory cancer was 1.56 (95% CI, 1.4–1.7). In the restricted data, the mean cumulative arsenic exposure was 5.4 mg/m^3^-years, the crude respiratory mortality rate per 1,000 person-years was 1.80, and the SMR was 1.87 (95% CI, 1.7–2.1).

SMRs increased with cumulative arsenic exposure and with arsenic concentration (Table 2). The SMR in the lowest concentration category was significantly elevated, suggesting a difference between respiratory cancer mortality rates for U.S. males and a hypothetical group of nonexposed workers. We fitted model 1 with continuous cumulative arsenic exposure and six categories of arsenic concentration and estimated respiratory cancer rates in a hypothetical group of nonexposed workers relative to the standard population. After adjustment for arsenic exposure, SMRs for nonexposed workers were 3.10 (95% CI, 2.3–4.2), 2.22 (95% CI, 1.6–3.1), and 1.44 (95% CI, 1.1–2.0) for calendar periods < 1960, 1960–69, and ≥1970, respectively, and the effect of U.S. birth relative to foreign birth was 0.67 (95% CI, 0.5–0.9). SMRs did not vary significantly with attained age. We included offset parameters fixed at the logarithms of these “nonexposed” SMRs to adjust for differences between “nonexposed” workers and the standard population. After adjustment, SMRs increased with cumulative arsenic exposure and with arsenic concentration, but at a lesser rate of increase (Table 2).

**Figure 1:**
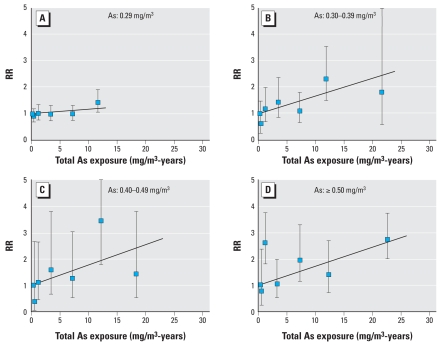
RRs of respiratory cancer mortality by categories of cumulative arsenic exposure (mg/m^3^-years) and arsenic concentration (mg/m^3^) relative to U.S. mortality rates for white males, adjusted to nonexposed workers, and fitted linear ERR models for cumulative arsenic exposure: 0.29 mg/m^3^ (*A*), 0.30–0.39 mg/m^3^ (*B*), 0.40–0.49 mg/m^3^ (*C*), and ≥0.50 mg/m^3^ (*D*). Estimates of the ERR per mg/m^3^-year and 95% CIs for the four concentration categories were as follows: A, 0.016 (–0.005 to 0.041); B, 0.067 (0.024 to 0.119); C, 0.077 (0.017 to 0.159); D, 0.072 (0.043 to 0.107).

Within each arsenic concentration category, RRs increased with cumulative arsenic exposure (Figure 1). Two characteristics in the figure are noteworthy. First, RRs by cumulative arsenic exposure were consistent with linearity within each concentration category. Tests of the null hypothesis of no departure from linearity were not rejected. Second, estimates of the slope parameters γ*_i_* (i.e., the ERR/mg/m^3^-year) varied significantly (*p*-value for homogeneity of slopes, *p* = 0.02) and generally increased, suggesting a greater exposure–response relationship with increasing arsenic concentration.

**Figure 2:**
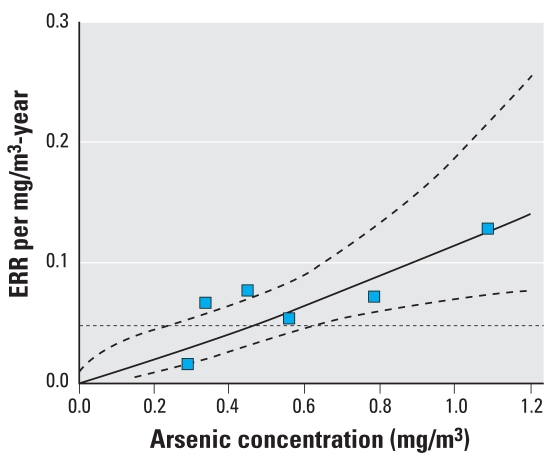
Estimates of ERR per mg/m^3^-year based on a linear RR model within six categories of arsenic concentration (square symbols), fitted model 2 (solid line), its pointwise, two-sided, Wald 95% CI (dashed lines), and model 2 omitting variation with concentration (dotted line; ERR/mg/m^3^/year = 0.04756).

The change in the ERR/mg/m^3^-year estimate (i.e., the slope) represents the effect modification by arsenic concentration of the relationship between respiratory cancer and cumulative arsenic exposure. Figure 2 plots the slope estimate by mean arsenic concentration for six concentration categories (square symbols, with CIs omitted for clarity), the fitted model 2 using continuous data (solid line), its pointwise 95% CI (dashed lines), and the model for no variation with concentration (model 2 with φ = 0; dotted line). The ERR/mg/m^3^-year estimate (β) was β = 0.115 (95% CI 0.07–0.19) (Table 3), and varied significantly with arsenic concentration (*p* < 0.01). After adjusting for cumulative arsenic exposure, the ERR/mg/m^3^-year increased with increasing concentration (estimate of φ was 1.123 (95% CI, 0.41–1.84). Results in the restricted data were similar.

Using model 2, we evaluated time since last arsenic exposure as a modifier of the arsenic exposure–response relationship. We focus on the restricted data, although we show results for all data for completeness. Although variations were not statistically significant, the cumulative arsenic exposure effect (β) decreased with time since last arsenic exposure by factors of 1.0, 0.8, and 0.2 for < 5, 5–14, and ≥15 years since last exposure, respectively (Table 3; change in deviance = 2.6 and *p* = 0.27 comparing models B0-R and T1-R; or change in deviance = 1.2 and *p* = 0.55 comparing models T2-R and T3-R). The arsenic concentration effect (φ) also varied with time since last exposure, but changes in deviance were smaller (Table 3; change in deviance = 1.9 and *p* = 0.39 comparing models B0-R and T2-R, or change in deviance = 0.5 and *p* = 0.78 comparing T1-R and T3-R).

In the restricted data, there was a suggestion of declining arsenic effects with attained age, but no preference for effect modification of age with either cumulative arsenic exposure or arsenic concentration.

## Discussion

In our study of copper smelter workers, RRs for respiratory cancer increased linearly with cumulative arsenic exposure within categories of arsenic concentration. In addition, we found a direct concentration effect, whereby the slope of the linear exposure–response relationship increased with arsenic concentration. This pattern implied that for equal cumulative arsenic exposure, the RR of respiratory cancer mortality was greater for cumulative arsenic exposure delivered at higher concentration for shorter duration compared with cumulative exposure delivered at lower concentration for longer duration. The increase in the exposure–response relationship within each arsenic concentration category occurred for the full range of concentrations experienced by smelter workers, suggesting that the direct concentration effect was not rate limited within this range.

One explanation for the impact of arsenic concentration on the association between cumulative arsenic exposure and respiratory cancer may be found in the concentration-dependent ability of cells to methylate and detoxify arsenic. Although human populations differ, most humans exposed to arsenic excrete 10–30% inorganic arsenic, 10–20% monomethylarsonic acid [MMA(V) and MMA(III)], and 60–80% dimethylarsinic acid [DMA(V) and DMA(III)]. The pentavalent metabolites MMA(V) and DMA(V) are generally considered less toxic than inorganic arsenite or arsenate. Trivalent methylated arsenic metabolites are more genotoxic than inorganic arsenite both *in vitro* and *in vivo* (Styblo et al. 2002). Although MMA(III) and DMA(III) can be measured in urine, epidemiologic studies to date have not measured trivalent arsenicals because of the preservation techniques required (Aposhian et al. 2000; Le et al. 2000; Mandal et al. 2001; Valenzuela et al. 2005). Epidemiologic studies have used the percent MMA of total inorganic arsenic and the MMA:DMA ratio as surrogate markers of methylation efficiency of ingested arsenic, with increased levels of excreted percent MMA and MMA:DMA ratio in urine indicating reduced methylation efficiency. Studies of arsenic in drinking water conducted in Taiwan, Argentina, Bangladesh, and certain areas of the United States have reported elevated risks of bladder and skin cancers associated with an increased percent MMA in urine, as well as a greater MMA:DMA ratio (Ahsan et al. 2007; Chen et al. 2003a, 2003b; Hsueh et al. 1997; Pu et al. 2007; Steinmaus et al. 2006; Yu et al. 2000). These findings are supported by toxicologic studies showing that methylated arsenicals, particularly MMA(III), have a greater affinity than the nonmethylated forms for protein binding to monothiol, dithiol, and trithiol sites, which can disrupt a number of processes, including gene transcription, glutathione synthesis, DNA methylation, DNA repair, cell division, generation of reactive oxygen species, and signal transduction pathways within cells (Cullen and Reimer 1989; Kitchin and Wallace 2008; Kumagai and Sumi 2007; Lin et al. 1999, 2001; Mass et al. 2001; Petrick et al. 2001; Styblo et al. 2000; Styblo and Thomas 1997). In addition, studies of urinary arsenic methylation patterns of healthy individuals exposed to arsenic in drinking water have shown increased percent MMA relative to total urinary arsenic (Hopenhayn-Rich et al. 1996b), whereas the percentage of inorganic arsenic and the MMA:DMA ratio decreased when the arsenic concentration in drinking water was reduced to lower levels (Hopenhayn-Rich et al. 1996a). These findings suggest that the mechanisms through which arsenic is methylated and excreted can become rate-limited at higher concentrations, perhaps through several steps including enzyme-catalyzed methylation of arsenic through *S*-adenosyl methionine, or glutathione synthesis (Waters et al. 2004).

The toxicologic effects of arsenic described above are multifaceted. However, toxicologic studies conducted to date support only the notion that concentration of exposure has a direct impact on the degree of toxicity and/or DNA damage acquired. Total cumulative arsenic exposure is a function of both concentration and duration of arsenic exposure. Our findings suggest that rates of arsenic biotrans-formation and excretion are concentration dependent. Toxicologic studies designed to compare cellular and genetic toxicity at a given total cumulative dose delivered at different dose rates are needed to enhance understanding of the mechanistic implications of our results.

Consistent with our findings, two epidemiologic studies have suggested that the toxic effects from cumulative arsenic exposure in drinking water are greater among individuals exposed at higher, as opposed to lower, concentrations. In a case–control study of arsenic-related skin lesions in a screened population in Bangladesh, about 70% of subjects consumed drinking water with arsenic concentrations > 50 μg/L (Rahman et al. 2006). For similar levels of cumulative arsenic exposure, the odds ratios were higher for subjects with older ages at first exposure compared with those younger at first exposure. Because older age at first exposure would reflect shorter duration of exposure, the results suggested higher risks for cumulative exposures with shorter durations and correspondingly higher concentrations, as we observed for lung cancer among smelter workers. In a case–control study of bladder cancer in New Hampshire, odds ratios among smokers increased with higher concentrations of toenail arsenic, with risks being greater among individuals with shorter (< 15 years) compared with longer residence in the current home (Karagas et al. 2004).

A threshold for the carcinogenic effects of arsenic in drinking water at about 150 μg/L, below which there is no risk from exposure, has been suggested (Lamm et al. 2006). Although studies of noncancer outcomes, such as arsenical dermatoses, cardiovascular disease, and cerebrovascular disease, do not support a threshold (Ahsan et al. 2006; Engel et al. 1994; Rahman et al. 2006; Yu et al. 2006), studies of cancer outcomes after relatively low levels of exposure have been limited by low power and potential exposure misclassification (Cantor and Lubin 2007).

Our results may inform the exposure threshold issue. In areas of Taiwan with endemic blackfoot disease, Bates et al. (1992) estimated that consuming drinking water with 470 μg/L arsenic resulted in a lifetime intake of 30,000 mg arsenic. Ingestion of drinking water with 150 μg/L arsenic therefore corresponds approximately to a lifetime intake of 9,600 mg arsenic. Measurement of urinary arsenic and its metabolites serves as a measure of absorption of inhaled arsenic through the respiratory tract, because arsenic excretion occurs mainly through the kidneys (World Health Organization 2001). In a study of smelter workers in Tacoma, Washington, investigators empirically derived the following relationship between inhaled and urinary arsenic levels: airborne arsenic in micrograms per cubic meter = 0.0064 (urine arsenic in micrograms per liter)^1.924^ (Enterline et al. 1987). In our data, mean airborne arsenic concentrations for the four categories in Figure 1 ranged from 0.29 to 0.65 mg/m^3^, and therefore correspond roughly to urinary arsenic levels from 263 to 400 μg/L. Assuming 2 L urine per day, 250 working days per year, no other sources of arsenic and no accumulation of arsenic in the body (Bates et al. 1992), the mean durations of exposure, which ranged from 9.7 to 12.1 years for the four categories, correspond to mean total arsenic intakes of 1,315 (= 263 × 2 × 250 × 10 year/1,000), 1,420, 1,649, and 1,996 mg arsenic, respectively. Thus, cumulative arsenic exposure in the smelter workers corresponded to < 20% of the lifetime arsenic intake at the postulated threshold. Our finding of a linear relationship for the RR of respiratory cancer mortality across the full range of cumulative arsenic exposures within each category of arsenic concentration argues against a threshold at 150 μg/L.

In summary, our cohort study of copper smelter workers revealed a linear relationship between cumulative arsenic exposure and respiratory cancer mortality within categories of arsenic concentration. In addition, we found a direct concentration effect on the exposure–response relationship, indicating that for a fixed level of cumulative arsenic exposure, inhalation of higher concentrations of arsenic over shorter durations was more deleterious than inhalation of lower concentrations over longer durations.

## Figures and Tables

**Table 1:** Descriptive statistics for the Montana cohort study of copper smelter workers.

Characteristic	All data	Restricted data^a^
No. of workers	8,014	7,887^b^
Person-years	256,850	144,851
Follow-up years	1938–1989	—
Mean cumulative arsenic exposure^c^ (mg/m^3^-years)	3.7	5.4
Mean airborne arsenic concentration^c^ (mg/m^3^)	0.35	0.36
Mean duration^c^ (years)	10.3	14.9
Respiratory cancer cases		
No.	446	261
Mean cumulative arsenic exposure (mg/m^3^-years)	7.0	10.4
Mean airborne arsenic concentration (mg/m^3^)	0.41	0.40
Mean duration (years)	17.4	26.0
Crude respiratory cancer mortality rate × 1,000	1.74	1.80

a Person-years in current and recent former (< 5 years) workers and workers with last employment at ≥ 55 years of age.

b Number of workers contributing person time.

c Person-years weighted mean.

**Table 2:** Summary data, respiratory cancer mortality rates, unadjusted and adjusted SMRs relative to U.S. white males by cumulative arsenic exposure (mg/m^3^-years) and arsenic concentration (mg/m^3^) for the Montana cohort study of copper smelter workers.

	Cumulative arsenic exposure (mg/m^3^-years)						Mean arsenic concentration (mg/m^3^)			
	< 0.75	0.75–1.99	2.0–4.9	5.0–9.9	10.0–14.9	≥ 15.0	0.29	0.30–0.39	0.40–0.49	≥ 0.50
Cases	62	96	74	83	84	47	239	74	29	104
Person-years	71424.0	66757.4	55332.2	39257.0	16804.7	7274.7	167583.2	39757.0	14853.6	34656.4
Mean	0.47	1.24	3.43	7.27	11.9	21.9	0.29	0.34	0.45	0.65
Crude rate × 1,000	0.87	1.44	1.34	2.11	5.00	6.46	1.43	1.86	1.95	3.00
SMR	0.97	1.50	1.35	1.49	2.40	3.62	1.30	1.61	1.81	2.59
95% CI	0.8–1.3	1.2–1.8	1.1–1.7	1.2–1.8	1.9–3.0	2.7–4.8	1.1–1.5	1.3–2.0	1.3–2.6	2.1–3.1
SMR^a^	0.84	1.28	1.08	1.11	1.68	2.35	1.02	1.32	1.47	2.00
90% CI	0.6–1.1	1.0–1.6	0.9–1.4	0.9–1.4	1.4–2.1	1.8–3.1	0.9–1.2	1.1–1.7	1.0–2.1	1.7–2.4

a SMRs adjusted for differences by calendar period and country of birth between rates of respiratory cancer in U.S. males and estimated respiratory cancer rates in a hypothetical group of nonexposed workers.

**Table 3:** Results of modeling the RR^a^ of respiratory cancer by cumulative arsenic exposure and arsenic concentration and variations by time since last exposure (TSLE) and attained age: Montana cohort study of copper smelter workers.

Model	β	TSLE (θ_TSLE_): < 5, 5–14, ≥ 15	φ	φ _TSLE_	Deviance^b^
B0^c^	0.115		1.123	—	
T1	0.120	1.00, 0.83, 1.20	1.153		0.5
T2	0.115			0.923, 1.278, 2.077	1.3
T3	0.095	1.00, 1.02, 2.52		0.723, 1.095, 2.661	3.7
B0-R^c^	0.083		0.822	—	
T1-R	0.102	1.00, 0.75, 0.18	0.848		2.6
T2-R	0.085			0.632, 1.111, 3.486	1.9
T3-R	0.095	1.00, 0.99, 0.11		0.739, 1.240, 17.53	3.1
Model	β	Age (θ_Age_): < 60, 60–69, ≥ 70	φ	φ _Age_	Deviance^b^

B0^c^	0.115		1.123	—	
A1	0.153	1.00, 0.88, 0.52	1.175		2.2
A2	0.115			1.285, 1.012, 1.187	0.2
A3	0.200	1.00, 0.67, 0.20		1.830, 1.153, 0.077	4.9
B0-R^c^	0.083		0.822	—	
A1-R	0.088	1.00, 0.88, 0.66	0.878		0.7
A2-R	0.082			1.118, 0.813, 0.678	0.3
A3-R	0.156	1.00, 0.64, 0.20		1.724, 1.001, –0.281	4.0

a RR model: RR = 1 + β *d* θ*_f_* exp{φ *_f_* ln(*c*)}, where *d* is cumulative arsenic exposure in mg/m3-years, and *c* is airborne arsenic concentration in mg/m^3^. Subscript *f* denotes categories of TSLE or attained age and represents multiple parameters either multiplying the ERR per mg/m^3^-year (β) or distinct categories for the effects of arsenic concentration.

b Change in deviance from the base model, B0. Difference in deviances provides a likelihood ratio test of the added parameters.

c Models fit to all cohort data or to restricted data, denoted by “R,” including person-time of workers with time since last exposure < 5 years or age last exposure ≥50. Estimates and 95% CIs: B0 parameters (β and φ), 0.115 (0.07–0.19) and 1.123 (0.41– 1.84); B0-R parameters, 0.083 (0.04–0.15) and 0.822 (0.01–0.63).
